# The reporting of studies conducted using observational routinely collected health data statement for pharmacoepidemiology (RECORD-PE)

**DOI:** 10.1136/bmj.k3532

**Published:** 2018-11-14

**Authors:** Sinéad M Langan, Sigrún AJ Schmidt, Kevin Wing, Vera Ehrenstein, Stuart G Nicholls, Kristian B Filion, Olaf Klungel, Irene Petersen, Henrik T Sorensen, William G Dixon, Astrid Guttmann, Katie Harron, Lars G Hemkens, David Moher, Sebastian Schneeweiss, Liam Smeeth, Miriam Sturkenboom, Erik von Elm, Shirley V Wang, Eric I Benchimol

**Affiliations:** 1 Faculty of Epidemiology and Population Health, London School of Hygiene and Tropical Medicine, London WC1E 7HT, UK; 2 Department of Clinical Epidemiology, Aarhus University, Aarhus, Denmark; 3 Ottawa Hospital Research Institute, Ottawa, ON, Canada;; 4 School of Epidemiology and Public Health, University of Ottawa, Ottawa, ON, Canada; 5 Departments of Medicine and of Epidemiology, Biostatistics, and Occupational Health, McGill University, Montreal, QC, Canada; 6 Centre for Clinical Epidemiology, Lady Davis Institute, Jewish General Hospital, Montreal, QC, Canada; 7 Division of Pharmacoepidemiology and Clinical Pharmacology, Utrecht Institute for Pharmaceutical Sciences, Utrecht University, Utrecht, Netherlands; 8 Department of Primary Care and Population Health, University College London, London, UK; 9 Arthritis Research UK Centre for Epidemiology, Division of Musculoskeletal and Dermatological Sciences, School of Biological Sciences, Manchester Academic Health Science Centre, University of Manchester, Manchester, UK; 10 Institute for Clinical Evaluative Sciences, Toronto, ON, Canada; 11 Hospital for Sick Children, Department of Paediatrics, University of Toronto, Toronto, ON, Canada; 12 ICH Population, Policy, and Practice Programme, University College London, Great Ormond Street Institute of Child Health, London, UK; 13 Basel Institute for Clinical Epidemiology and Biostatistics, Department of Clinical Research, University Hospital Basel, University of Basel, Basel, Switzerland; 14 Division of Pharmacoepidemiology and Pharmacoeconomics, Department of Medicine, Brigham and Women’s Hospital and Harvard Medical School, Boston, MA, USA; 15 Julius Global Health, University Medical Center Utrecht, Utrecht, Netherlands; 16 Cochrane Switzerland, Institute of Social and Preventive Medicine, University of Lausanne, Lausanne, Switzerland; 17 Department of Pediatrics and School of Epidemiology and Public Health, University of Ottawa, Ottawa, ON, Canada; 18 Children’s Hospital of Eastern Ontario Research Institute, Ottawa, ON, Canada

## Abstract

In pharmacoepidemiology, routinely collected data from electronic health records (including primary care databases, registries, and administrative healthcare claims) are a resource for research evaluating the real world effectiveness and safety of medicines. Currently available guidelines for the reporting of research using non-randomised, routinely collected data—specifically the REporting of studies Conducted using Observational Routinely collected health Data (RECORD) and the Strengthening the Reporting of OBservational studies in Epidemiology (STROBE) statements—do not capture the complexity of pharmacoepidemiological research. We have therefore extended the RECORD statement to include reporting guidelines specific to pharmacoepidemiological research (RECORD-PE). This article includes the RECORD-PE checklist (also available on www.record-statement.org) and explains each checklist item with examples of good reporting. We anticipate that increasing use of the RECORD-PE guidelines by researchers and endorsement and adherence by journal editors will improve the standards of reporting of pharmacoepidemiological research undertaken using routinely collected data. This improved transparency will benefit the research community, patient care, and ultimately improve public health.

Routinely collected health data are a by-product of the daily operations of healthcare systems, collected independently of specific a priori research questions.^1^ ^2^ A broad range of sources (eg, disease registries, health administrative data, quality/safety surveillance databases, electronic health records, and pharmacy data) contain routinely collected data and have both drug exposure and clinical outcomes that are of potential use in pharmacoepidemiology.^3^ ^4^

In pharmacoepidemiology, routinely collected health data are a broadly accepted, necessary, and cost effective resource widely used for evaluating the real world effectiveness and safety of medicines. Studies conducted with routinely collected data are necessary for many reasons. Clinical trials might not be available, or ethical, and could have limitations owing to restrictive inclusion and exclusion criteria. Primary data collection could be costly or infeasible, have limited statistical power to detect safety events, or have durations that prevent the assessment of long term safety outcomes. In many cases, routinely collected health data can be used to provide timely answers and reduce waste in biomedical research when analysing important and novel healthcare issues. The use of routinely collected health data not only leverages existing investment but also could reduce the need for additional investment in de novo data collection.^5^ ^6^ Research based on real world evidence, such as routinely collected data, has been conducted on health system planning and evaluation, drug utilisation, comparative drug effectiveness, epidemiological surveillance, and postmarketing drug surveillance (phase IV studies).^7^ ^8^ ^9^

Although routinely collected health data are commonly used in pharmacoepidemiological research, these studies are often suboptimally reported.^10^ ^11^ ^12^ Reporting guidelines have been developed for a range of study designs, and represent a minimum standard or items that should be reported in academic manuscripts.^13^ ^14^ The main purposes of reporting guidelines are to ensure that readers can easily determine the research question, the methodology used, and the study findings; facilitate understanding of study strengths and limitations, specifically providing insight regarding possible biases; and facilitate replication. Reporting guidelines can also indirectly improve the quality of research by indicating which items to address during study design.^15^ ^16^

The RECORD (REporting of studies Conducted using Observational Routinely collected Data) guideline represents the current best practice standard for the reporting of research using non-randomised routinely collected health data. The guideline was the product of an international collaboration focused on improving the reporting of observational studies using routinely collected data.^1^ ^17^ RECORD consists of a checklist of 13 items that supplement or modify the earlier best practice guideline, STROBE (STrengthening the Reporting of OBservational studies in Epidemiology), which focused on the reporting of observational studies.^1^ ^18^ The RECORD statement was informed by a systematic review that highlighted major deficiencies in the reporting of studies using routinely collected health data.^11^ Since its publication, RECORD has been endorsed by more than 20 major journals (for more information, see www.record-statement.org).

However, the methodological complexity of pharmacoepidemiological research means that certain reporting requirements are beyond the scope of either RECORD or STROBE. Here, we aimed to extend the RECORD statement to include reporting guidelines specific to pharmacoepidemiological research—that is, the reporting of research focusing on the uses and effects of drugs.^19^ This initiative is complementary to existing guidance in the field that mainly focuses on methods for doing (instead of reporting) pharmacoepidemiological research and evaluating the quality of published papers.^20^ ^21^ We welcome global community engagement in this endeavour and comments from interested parties by email as these guidelines will be updated periodically.

**Summary points**

The RECORD reporting guidelines represent the current best practice standard to ensure the clarity and completeness of reporting of non-interventional research using routinely collected health data
The RECORD-PE statement was derived by use of rigorous methodology and endorsed by the International Society for Pharmacoepidemiology. It is intended to act as a guideline to improve the reporting of pharmacoepidemiological research undertaken using routinely collected health data
The 15 item checklist should be used in parallel with the RECORD and STROBE guidelines to ensure transparent reporting of pharmacoepidemiology studies using routinely collected health data

## The RECORD for Pharmacoepidemiology (RECORD-PE) checklist

### Creation and development of the checklist

We convened a group of international experts in pharmacoepidemiology, routinely collected health data research, reporting guidelines, journalology (that is, the science of publication practices), the joint International Society for Pharmacoepidemiology/International Society for Pharmacoeconomics and Outcomes Research consensus paper on reporting requirements to make database studies reproducible,^22^ and knowledge users to adapt RECORD for non-interventional pharmacoepidemiological research (RECORD-PE), as presented below. Draft items to be considered in a RECORD-PE checklist were proposed by authors and considered during regular teleconferences and electronic communication, resulting in a draft checklist. A face-to-face meeting was then held in Montreal, Canada, on 25 August 2017. At this meeting, attendees voted on the inclusion of proposed statements and the appropriate wording of these statements, using the approach previously described for the creation of the RECORD statement.^17^ Items were included in the checklist if more than 80% of participants agreed on the concept, wording, and message of the item. The draft manuscript and checklist were subsequently revised and circulated to all authors and the RECORD steering committee for comment and approval. It was also circulated to the members of the International Society for Pharmacoepidemiology for comment after completion of the draft and revised accordingly.

### RECORD-PE checklist items

Table 1 shows the complete RECORD-PE checklist, which is organised according to standard manuscript sections and follows the conventions set out in STROBE (and subsequently RECORD).^1^ ^18^ The checklist consists of 15 additional items, of which 13 focus on the methods section. Because this checklist is an extension of RECORD, which in turn is an extension of available STROBE items, the statements specific to pharmacoepidemiology are presented next to corresponding STROBE and RECORD checklist items. STROBE additionally has specific checklists for study designs including cohort, cross sectional, and case-control studies. For RECORD and RECORD-PE, we have extended the general STROBE checklist.^10^ ^18^ Authors will be expected to consider each checklist item when drafting their manuscript and include items in their manuscript submissions. Below, we provide explanatory text for each RECORD-PE checklist item, organised by manuscript section, and provide a glossary in supplementary table 1. 

**Table 1:** The RECORD statement for pharmacoepidemiology (RECORD-PE) checklist of items, extended from the STROBE and RECORD statements,^1^ ^18^ which should be reported in non-interventional pharmacoepidemiological studies using routinely collected health data

Item No	STROBE items	RECORD items	RECORD-PE items	Page No
**Title and abstract **				
1	(a) Indicate the study’s design with a commonly used term in the title or the abstract. (b) Provide in the abstract an informative and balanced summary of what was done and what was found.	1.1: The type of data used should be specified in the title or abstract. When possible, the name of the databases used should be included. 1.2: If applicable, the geographical region and timeframe within which the study took place should be reported in the title or abstract. 1.3: If linkage between databases was conducted for the study, this should be clearly stated in the title or abstract.	—	
**Introduction **				
Background rationale				
2	Explain the scientific background and rationale for the investigation being reported.	—	—	
Objectives				
3	State specific objectives, including any prespecified hypotheses.	—	—	
**Methods **				
Study design				
4	Present key elements of study design early in the paper.	—	4.a: Include details of the specific study design (and its features) and report the use of multiple designs if used. 4.b: The use of a diagram(s) is recommended to illustrate key aspects of the study design(s), including exposure, washout, lag and observation periods, and covariate definitions as relevant.	
Setting				
5	Describe the setting, locations, and relevant dates, including periods of recruitment, exposure, follow-up, and data collection.	—	—	
Participants				
6	(a) Cohort study—give the eligibility criteria, and the sources and methods of selection of participants. Describe methods of follow-up. Case-control study—give the eligibility criteria, and the sources and methods of case ascertainment and control selection. Give the rationale for the choice of cases and controls. Cross sectional study—give the eligibility criteria, and the sources and methods of selection of participants. (b) Cohort study—for matched studies, give matching criteria and number of exposed and unexposed. Case-control study—for matched studies, give matching criteria and the number of controls per case.	6.1: The methods of study population selection (such as codes or algorithms used to identify participants) should be listed in detail. If this is not possible, an explanation should be provided. 6.2: Any validation studies of the codes or algorithms used to select the population should be referenced. If validation was conducted for this study and not published elsewhere, detailed methods and results should be provided. 6.3: If the study involved linkage of databases, consider use of a flow diagram or other graphical display to demonstrate the data linkage process, including the number of individuals with linked data at each stage.	6.1.a: Describe the study entry criteria and the order in which these criteria were applied to identify the study population. Specify whether only users with a specific indication were included and whether patients were allowed to enter the study population once or if multiple entries were permitted. See explanatory document for guidance related to matched designs.	
Variables				
7	Clearly define all outcomes, exposures, predictors, potential confounders, and effect modifiers. Give diagnostic criteria, if applicable.	7.1: A complete list of codes and algorithms used to classify exposures, outcomes, confounders, and effect modifiers should be provided. If these cannot be reported, an explanation should be provided.	7.1.a: Describe how the drug exposure definition was developed. 7.1.b: Specify the data sources from which drug exposure information for individuals was obtained. 7.1.c: Describe the time window(s) during which an individual is considered exposed to the drug(s). The rationale for selecting a particular time window should be provided. The extent of potential left truncation or left censoring should be specified. 7.1.d: Justify how events are attributed to current, prior, ever, or cumulative drug exposure. 7.1.e: When examining drug dose and risk attribution, describe how current, historical or time on therapy are considered. 7.1.f: Use of any comparator groups should be outlined and justified. 7.1.g: Outline the approach used to handle individuals with more than one relevant drug exposure during the study period.	
Data sources/measurement				
8	For each variable of interest, give sources of data and details of methods of assessment (measurement). Describe comparability of assessment methods if there is more than one group.	—	8.a: Describe the healthcare system and mechanisms for generating the drug exposure records. Specify the care setting in which the drug(s) of interest was prescribed.	
Bias				
9	Describe any efforts to address potential sources of bias.	—	—	
Study size				
10	Explain how the study size was arrived at.	—	—	
Quantitative variables				
11	Explain how quantitative variables were handled in the analyses. If applicable, describe which groupings were chosen, and why.	—	—	
Statistical methods				
12	(a) Describe all statistical methods, including those used to control for confounding. (b) Describe any methods used to examine subgroups and interactions. (c) Explain how missing data were addressed. (d) Cohort study—if applicable, explain how loss to follow-up was addressed. Case-control study—if applicable, explain how matching of cases and controls was addressed. Cross sectional study—if applicable, describe analytical methods taking account of sampling strategy. (e) Describe any sensitivity analyses.	—	12.1.a: Describe the methods used to evaluate whether the assumptions have been met. 12.1.b: Describe and justify the use of multiple designs, design features, or analytical approaches.	
Data access and cleaning methods				
12	—	12.1: Authors should describe the extent to which the investigators had access to the database population used to create the study population. 12.2: Authors should provide information on the data cleaning methods used in the study.	—	
Linkage				
12	—	12.3: State whether the study included person level, institutional level, or other data linkage across two or more databases. The methods of linkage and methods of linkage quality evaluation should be provided.	—	
**Results **				
Participants				
13	(a) Report the numbers of individuals at each stage of the study (eg, numbers potentially eligible, examined for eligibility, confirmed eligible, included in the study, completing follow-up, and analysed). (b) Give reasons for non-participation at each stage. (c) Consider use of a flow diagram.	13.1: Describe in detail the selection of the individuals included in the study (that is, study population selection) including filtering based on data quality, data availability, and linkage. The selection of included individuals can be described in the text or by means of the study flow diagram.	—	
Descriptive data				
14	(a) Give characteristics of study participants (eg, demographic, clinical, social) and information on exposures and potential confounders. (b) Indicate the number of participants with missing data for each variable of interest. (c) Cohort study—summarise follow-up time (eg, average and total amount).	—	—	
Outcome data				
15	Cohort study—report numbers of outcome events or summary measures over time. Case-control study—report numbers in each exposure category, or summary measures of exposure. Cross sectional study—report numbers of outcome events or summary measures.	—	—	
Main results				
16	(a) Give unadjusted estimates and, if applicable, confounder adjusted estimates and their precision (eg, 95% confidence intervals). Make clear which confounders were adjusted for and why they were included. (b) Report category boundaries when continuous variables are categorised. (c) If relevant, consider translating estimates of relative risk into absolute risk for a meaningful time period.	—	—	
Other analyses				
17	Report other analyses done—eg, analyses of subgroups and interactions, and sensitivity analyses.	—	—	
**Discussion **				
Key results				
18	Summarise key results with reference to study objectives.	—	—	
Limitations				
19	Discuss limitations of the study, taking into account sources of potential bias or imprecision. Discuss both direction and magnitude of any potential bias.	19.1: Discuss the implications of using data that were not created or collected to answer the specific research question(s). Include discussion of misclassification bias, unmeasured confounding, missing data, and changing eligibility over time, as they pertain to the study being reported.	19.1.a: Describe the degree to which the chosen database(s) adequately captures the drug exposure(s) of interest.	
Interpretation				
20	Give a cautious overall interpretation of results considering objectives, limitations, multiplicity of analyses, results from similar studies, and other relevant evidence.	—	20.a: Discuss the potential for confounding by indication, contraindication or disease severity or selection bias (healthy adherer/sick stopper) as alternative explanations for the study findings when relevant.	
Generalisability				
21	Discuss the generalisability (external validity) of the study results.	—	—	
**Other information **				
Funding				
22	Give the source of funding and the role of the funders for the present study and, if applicable, for the original study on which the present article is based.	—	—	
Accessibility of protocol, raw data, and programming code				
22	—	22.1: Authors should provide information on how to access any supplemental information such as the study protocol, raw data, or programming code.	—	

RECORD=reporting of studies conducted using observational routinely collected data; RECORD-PE=RECORD for pharmacoepidemiological research; STROBE=strengthening the reporting of observational studies in epidemiology. This table can be downloaded as a separate document in the web appendix; page numbers can be added electronically to the PDF document.

For content sufficiently covered under STROBE or RECORD, no additional items are provided, although explanatory text regarding particular aspects that might be more pertinent to pharmacoepidemiological studies is provided. All relevant explanations are presented under the respective RECORD-PE item or article section. The RECORD-PE statement is intended for use only in reporting on pharmacoepidemiology studies conducted with routinely collected health data, and represents a minimum standard of reporting for such research in published papers. Such studies include investigation of the use, effectiveness, and safety of drugs or drug eluting devices (eg, drug eluting stents) used in clinical practice. In addition to the widely accepted uses of routinely collected health data for pharmacoepidemiology, in recent years, the concept has emerged that cohort studies of interventions using such data could also be considered attempts to emulate a target trial of the intervention of interest. This concept has been considered helpful within epidemiology and pharmacoepidemiology.^23^ ^24^ Routinely collected health data can also help studies with baseline randomisation or pragmatic trials, because the data are frequently collected as part of routine care or health system administration; however, guidance for the reporting of pragmatic trials or trials using these data is beyond the scope of RECORD-PE.

## Keywords and medical subject headings terms

The STROBE and RECORD statements do not address the use of specific keywords or medical subject headings (MeSH) terms to identify studies using routinely collected health data.^1^ ^25^ There are currently no specific MeSH terms to identify these studies, and researchers use a range of search terms to identify these studies, which is a clear limitation in terms of undertaking systematic reviews and meta-epidemiological research and highlights a need for future research focus.^11^

## Title and abstract

No items specific to the RECORD-PE guidelines are needed in addition to the STROBE and RECORD items for the title and abstract. STROBE guidelines advise that an abstract should provide “an informative and balanced summary of what was done and found.”^18^ Providing such a summary in the abstract highlights that the study is a pharmacoepidemiological study, and details the research question, the approach taken, and the study findings. Because screening of titles and abstracts is a key step in knowledge syntheses (eg, scoping reviews, systematic reviews), clarity in wording the title and abstract will facilitate appropriate reuse of research findings, thus reducing the waste of research resources. In describing the conduct of a study using routinely collected health data, the RECORD guideline items recommend reporting the type of data used and the name of the database(s), including highlighting whether linked data were used; these specifications are also directly relevant to pharmacoepidemiological studies.^1^

## Introduction

No items specific to the RECORD-PE guidelines are needed in addition to the STROBE and RECORD items for the introduction section. The STROBE guidelines advise authors to detail “specific objectives, including any prespecified hypotheses.”^18^ The RECORD explanatory paper further recommends that authors be explicit about whether analyses were exploratory or confirmatory, post hoc or prespecified, or a mixture of these characteristics. Authors should highlight how interested readers can access the study protocol. These recommendations are needed to enable stakeholders to interpret pharmacoepidemiological studies.

## Methods (study design)

### RECORD-PE item 4.a

Include details of the specific study design (and its features) and report the use of multiple designs if used.

#### Explanation

STROBE recommends that researchers present key elements of the study design early in the paper.^18^ Because routinely collected health data are typically collected in advance of undertaking a study, researchers can theoretically use a range of study designs (eg, self controlled case-series, cohort or case-control studies) or design features (eg, new user designs) depending on the research question. Use of a range of study designs within papers was not directly considered by RECORD. Two aspects of pharmacoepidemiological research warrant an extension to the STROBE statement. Firstly, researchers in the field often use specific study design features (eg, the active comparator new user design) not covered by existing STROBE guidance. Secondly, it is common to use more than one such design or design feature in one publication. Readers should be able to determine which study designs or design features were used. This information will facilitate those readers interested in replicating the methods used.

Study authors should describe such study designs or design features with as much detail as is necessary to make clear to readers what the design involved. If using multiple study designs or design features, authors should comment on which was used for the primary analysis. Authors also should comment on and justify deviations from any study protocol or explicitly state that there was no changes from the protocol.

#### Examples

*Specific design feature (active comparator new user design)*—“A new-user cohort design was used to compare patients initiating dabigatran or rivaroxaban at standard doses for treatment of non valvular AF [atrial fibrillation]. We identified all patients with any inpatient or outpatient diagnoses of AF or atrial flutter, based on *International Classification of Diseases, Ninth Revision (ICD-9)*, coding, who filled their first prescription for either drug from November 4, 2011, when rivaroxaban was approved for AF in the United States, through June 30, 2014. Patients were excluded if they had less than 6 months of enrolment in Medicare Parts A, B, and D, were younger than 65 years, had received prior treatment with warfarin or any NOAC [novel oral anticoagulant], resided in a skilled nursing facility or nursing home, or were receiving hospice care on the date of their cohort-qualifying prescription (index date) . . . Because our purpose was to directly compare dabigatran and rivaroxaban, we did not include a warfarin-treated cohort.”^26^

*Specific design feature (interrupted time series analysis)*—“To estimate trend changes in antibiotic prescribing over time, we used segmented linear regression analysis of interrupted time series data, a common quasi-experimental method to assess trend changes after clearly defined events. Separately for each birth week cohort, we estimated the 1-year risk of redeeming at least 1 prescription for an antibiotic, with subanalysis for amoxicillin and penicillin V.”^27^

*Specific design feature (drug utilisation/evaluation of the effectiveness of risk minimisation interventions)*—“Following a centralised authorisation within the European Union, on 1 August 2011, dabigatran was marketed in two doses (either 150 or 110 mg bid) for stroke prevention in patients with NVAF [non-valvular atrial fibrillation] and having one or more stroke risk factors. . . . Following early post-marketing reports of bleedings, cautionary recommendations were issued by regulatory authorities. In the safety update from the European Medicines Agency (EMA) (18 November 2011), it was recommended that low doses should be prescribed to elderly patients. Also, this update emphasised the importance of monitoring of renal function, in particular in patients over 75 years. The impact of this safety update, as well as the features of the framework previously described, is assessed in the case study described below.”^28^

### RECORD-PE item 4.b

The use of a diagram(s) is recommended to illustrate key aspects of the study design(s), including exposure, washout, lag and observation periods, and covariate definitions as relevant.

#### Explanation

We recommend the inclusion of a diagram or figure that illustrates the overall study design or timeline for patient inclusion (including key study aspects such as prescription start and end, risk periods, exposed periods, unexposed periods, grace periods, induction periods, and washout periods). Exposure periods are complex in pharmacoepidemiology and are often difficult for readers to follow; this item was not specifically recommended by RECORD. If more than one type of design or analysis is included in the study, a diagram for each is recommended. This allows potentially complex analysis designs, including multiple at-risk periods between or within patients, to be visually summarised in a way that can prevent misinterpretation of paragraphs of text describing the design and implementation.

#### Examples

**Fig 1:**
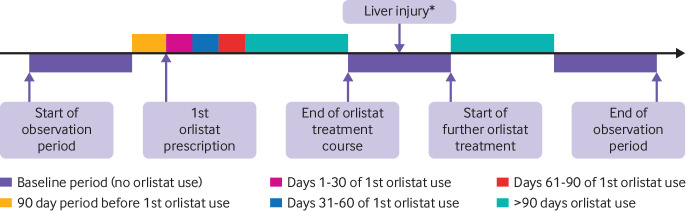
Example of a diagram showing a typical timeline for a patient. *Liver injury could occur at any point during observation period. Adapted from Douglas et al^29^ with permission

*Illustration of exposure assessment periods in self controlled case series studies*—a paper by Douglas and colleagues^29^ relates to the use of orlistat and risk of acute liver injury and contains a figure describing a typical timeline for a patient in the study (fig 1). The study used a self controlled case series design, and the diagram provides an example of the distribution of unexposed and exposed periods for one patient (baseline, pretreatment, and multiple time periods of orlistat exposure) and highlights the liver injury risk periods.

**Fig 2:**
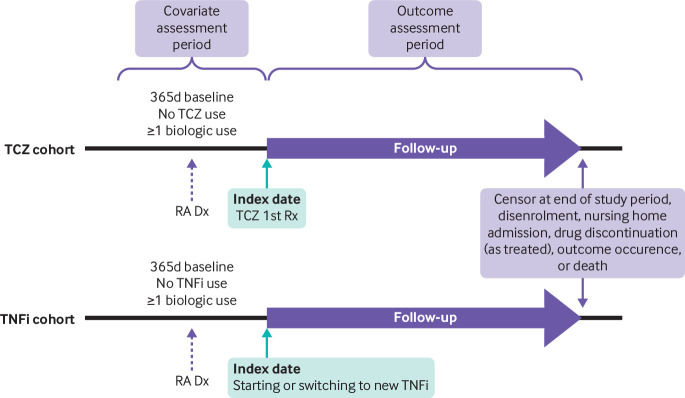
Example of a diagram illustrating cohort entry criteria, exposure assessment, and censoring events including time periods. RA=rheumatoid arthritis; TCZ=tocilizumab; TNFi=tumour necrosis factor inhibitors; Dx=diagnosis; Rx=prescription. Adapted from Kim et al^30^ with permission

*Illustration of exposure assessment periods in cohort studies*—this study by Kim and colleagues^30^ on tolciluzimab use and the risk for cardiovascular events describes an active comparator new user comparing two biological medicines to treat rheumatoid arthritis (fig 2). The figure shows two key inclusion criteria (use of ≥1 biological medicine and a diagnosis of rheumatoid arthritis without prior exposure to the specific drug), and the follow-up periods for the two exposure groups, including washout windows. Time periods are clearly marked and censoring events described.

## Methods (setting)

### RECORD-PE items

No additional RECORD-PE items are needed to broaden the existing STROBE items.

#### Explanation

As discussed in the RECORD explanatory document, readers need to understand both the reasons and context of data collection to be able assess the potential for information bias, for example, were the data collected for clinical care or billing purposes. Readers also should be able to determine whether the population in the database represents the source population in order to evaluate the generalisability of findings.

## Methods (participants)

### RECORD-PE item 6.1.a

Describe the study entry criteria and the order in which these criteria were applied to identify the study population. Specify whether only users with a specific indication were included and whether patients were allowed to enter the study population once or if multiple entries were permitted. See below for guidance related to matched designs.

#### Explanation

When patients are included in a study on the basis of their exposure status to one (or more) drugs, there are likely to be several ways of defining the entry criteria; hence an extension to RECORD is required. We refer here to three levels of population hierarchy described in detail in the RECORD explanatory text—namely, the source population, database population, and study population. In many Scandinavian databases, the source population and database population might represent the same individuals because they comprise the entire population of the specific country.^1^ The database population is derived from the study population and contains people who meet eligibility criteria (eg, in the case of primary care databases, they are in primary care practices and have not opted out of inclusion in the database). It is important to provide details of the inclusion and exclusion criteria applied to identify the study population, which includes clearly specification of how exposure status and other eligibility criteria are defined. Authors should also be clear whether the exclusion criteria are applied before or after selection of study entry dates. Reporting these details would greatly enhance study reproducibility and ability to evaluate the relevance and validity of findings.

A detailed description of matching procedures should be provided. For control sampling, the time axis on which the risk set or incidence density sampling was conducted should also be reported. The procedure for handling cases without eligible controls should be explained (eg*,* loosening of matching criteria, exclusion). A description should further include whether frequency or individual matching was used, whether matching was done with or without replacement, and the algorithm used (eg, greedy *v* nearest neighbour matching).

#### Examples of selection of the population

A study by van Staa and colleagues included users of oral corticosteroids who were defined as “permanently registered patients aged 18 years or older who received one or more prescriptions for oral corticosteroids during the period of time from the enrolment date in their practice in GPRD up to the end of the study (December 1997).”^31^

Shin and colleagues report: “The index date for cases was defined by the day of follow-up on which hospital admission occurred. For each case, up to 10 controls were randomly selected using risk set sampling, with controls matched on sex, age (±1 year), cohort entry date (±90 days), and follow-up duration; for one case, the age caliper was widened to 2 years to identify an eligible control.”^32^

## Methods (variables)

### RECORD-PE item 7.1.a

Describe how the drug exposure definition was developed.

#### Explanation

Authors should specify clearly how drug exposure code lists were obtained. This information could include which dictionary was searched (eg, the Anatomical Therapeutic Chemical (ATC) classification, or database or country specific codes such as the National Drug Codes in the US)^33^ and how these dictionaries or data sources were searched (eg, automated or manual), as well as which drug substance name(s) and what route of administration was used for the search and which ATC classification level was applied. This level of detail allows readers to interpret the completeness and veracity of the exposure definition and permits replication of study findings, and goes beyond the detail of RECORD.

#### Example

*Defining the drug exposure definition*—“The main exposures evaluated were first-trimester exposure to any antidepressants (medications with Anatomical Therapeutic Chemical Classification [ATC] codes beginning with N06A) and selective serotonin reuptake inhibitors (SSRIs; medications with ATC codes beginning with N06AB). Exposure was defined according to two sources of information: maternal self-reports (available for offspring born between 1996 and 2012) and dispensation records (available for both parents of offspring born between 2006 and 2012). Information about maternal *self-reported* medication use during the first trimester of pregnancy came from the Medical Birth Register . . . Information about medication use based on *dispensation* records came from the Prescribed Drug Register, which covers all medication dispensations and accompanying prescriptions written in Sweden since July 2005.”^34^

### RECORD-PE item 7.1.b

Specify the data sources from which drug exposure information for individuals was obtained.

#### Explanation

Authors should make clear what the data source is and whether the electronic records represent issued prescriptions from electronic health records or redeemed prescriptions. Readers also need to understand whether a database contains information on reimbursed prescriptions, out-of-network dispensations, drugs directly dispensed by healthcare providers (samples), or over-the-counter drug use and the completeness of these variables.

#### Example

*Specifying the data sources for drug exposure identification*—“In Denmark, the study population included users of OHAs [oral hypoglycaemic agents] identified in the Aarhus University Prescription Database (AUPD). The database’s catchment area covers the North and Central Regions of Denmark (hereafter referred to as ‘northern Denmark’), with a combined population in mid-2010 of 1.8 million persons, which is about one-third of the Danish population. AUPD captures reimbursed prescriptions redeemed in the regions’ outpatient pharmacies since 1998. In the UK, OHA users were identified from the General Practice Research Database (GPRD), currently also known as the Clinical Practice Research Datalink.”^35^

### RECORD-PE item 7.1.c

Describe the time window(s) during which an individual is considered exposed to the drug(s). The rationale for selecting a particular time window should be provided. The extent of potential left truncation or left censoring should be specified.

#### Explanation

The time of exposure can be, for instance, the number of days after the start of a first prescription (see also recommendation for figure in RECORD-PE item 4). The number of days may be derived from the number of tablets prescribed, the number of recorded refills, or the number of tablets taken per day for the stated or assumed indication. Frequently, for researchers using routinely collected health data, there might be no access to data regarding the instructions for taking the medicine. Readers should be able to know whether duration was assumed, based on usual prescribing or directly ascertained from instructions. A description of specific variables generated in association with drug exposure also should be provided. These could include variables capturing information related to dosage information or the total number of prescriptions redeemed within a defined period. Examples include duration, cumulative dose, and recency (that is, current, new, recent, former use). Authors should specify whether only initiators or both initiators and prevalent users are included. They should clearly outline whether they included new users and treatment naive new users by defining the required washout period before a patient is categorised as new user (eg, a new user, by contrast to a treatment naive new user, could be a re-initiator). Authors should detail assumptions about the prescribed daily dose (if not recorded), the duration of prescription coverage, and the length of grace periods used in defining characteristics such as switching, discontinuation, persistence, and adherence.^36^

To account for variation in refill behaviour, refills that are sufficiently close together usually are considered to represent continuous use. A drug is often considered discontinued, in the absence of a new refill, if a prespecified time interval goes past the assumed expiration date supplied in a given prescription (based on the estimated number of days prescribed). This issue could be important in systems such as electronic health records, which have variable coding dates. Readers may want to consult recently published recommendations on how to compute such durations of exposure and how to report methodology.^37^ The definition of the exposed period also can be used to assess the outcome of discontinuation. Because prescription or redemption records are an imperfect measure of true drug intake, the algorithms and assumptions used by authors to define exposed time should be reported. Different definitions of the exposed period can be examined by researchers in sensitivity analyses, which should be reported in the manuscript or appendices.

In routinely collected health data, issues of left or right truncation and censoring might also affect the definition of drug exposure and outcome data, and might result in important misclassification and bias—hence, these issues should be reported in publications of routinely collected health data.^38^ For example, right truncation could exist in an electronic health records setting because patients transfer between practices or within administrative systems if they become ineligible for insurance coverage; decisions around these aspects should be made clear to the reader.

#### Examples of defining exposure time windows

Patorno and colleagues report: “Exposure was defined as at least one filled prescription for lithium during the first trimester (first 90 days after the date of the last menstrual period). The primary reference group included women with no lithium or lamotrigine dispensings during the 3 months before the start of pregnancy or during the first trimester. The criterion of no dispensing during the 3 months before the start of pregnancy was imposed to avoid misclassifying as unexposed women who still had medications from an earlier filling available at the start of pregnancy.”^39^

Larivée and colleagues report: “As is true with most healthcare databases, data are left truncated, resulting in the incomplete capture of medical history and previous use of medications. This issue is particularly important in insurance databases, where no information is available outside of the coverage period, and databases such as US Medicare, which only cover patients aged 65 years or older. This truncation is partially mitigated in the CPRD [Clinical Practice Research Datalink] by the transfer of patient records from one practice to another when patients change practices, but such transfers are only feasible between practices that use the same software and it is not possible to link patient records across practices.”^40^

### RECORD-PE item 7.1.d

Justify how events are attributed to current, prior, ever, or cumulative drug exposure.

#### Explanation

In pharmacoepidemiological studies, it is common to compare rates of adverse events between two drug groups or two or more periods. The adverse event rate is defined as the number of adverse events divided by the total time at risk for a given exposure. It is important to consider and report transparently how the time at risk is defined. The definition of time at risk depends on the pharmacokinetic properties of the drug, the nature of the endpoint of interest, patient related factors, and the plausible hypothesis about the induction period linking the drug and the endpoint. Outcomes can be attributed to drug exposure anywhere along the spectrum from “currently exposed” to “ever exposed” when a binary exposure variable is considered. Another risk attribution model within this spectrum is “on drug plus a lag window.” In this model, an event can be attributed to treatment for a given time period beyond drug discontinuation, thereby allowing time for the drug to continue having a residual effect within the body, or for delayed presentation of the outcome. Different risk attribution models can lead to different conclusions based on the same data. This issue has led to guidelines for rheumatology biological medicine registers, suggesting that research groups use similar risk attribution models when addressing the same research question, to increase comparability of findings.^41^ When defining the risk attribution model, researchers may also consider the possibility of protopathic bias, for example, starting a drug to treat early symptoms of the undiagnosed outcome.^42^ If protopathic bias is possible, authors should describe it in their manuscript.

#### Example

*Describing how events are attributed to drug exposure*—[In the] “statistical analysis TB [tuberculosis] cases were attributed to anti-TNF [tumour necrosis factor] therapy using two different models: “on drug” (if the patient was actively receiving that drug at the time of diagnosis) and “most recent drug.”^43^

### RECORD-PE item 7.1.e

When examining drug dose and risk attribution, describe how current treatment, historical treatment, or time on treatment are considered.

#### Explanation

The risk of an adverse event could be influenced by current or historical treatment. Therefore, researchers should look at how current and past drug exposure are considered in analyses. Modelling only current use, either as a binary variable or as current dose, assumes that previous use has no effect on the outcome of interest. Recent use, such as exposure in the past 30 days, allows historical exposure to be considered, but assumes that exposure 29 days ago was important but exposure 31 days ago was not. Selection of an appropriate risk window varies according to the research question and the biological mechanism through which the exposure might lead to the outcome. For example, historical drug exposure is unlikely to influence a hypersensitivity reaction today, while drug exposure months or years ago could contribute to current risk of malignancy.^44^ Complex models, such as the weighted cumulative exposure model, allow history of drug use to be modelled flexibly up to the time point when risk is assessed.^45^ Although no model is perfect, researchers should consider and report how past exposure was taken into consideration. The approach for handling individuals who were exposed to more than one of the drug exposures of interest during the study period also should be outlined and authors will want to report their approach to time varying confounding. Authors might also want to directly address the issue of depletion of susceptible people or healthy adherer bias.^46^ ^47^

#### Examples describing how current and historical exposures are considered

Movahedi and colleagues report: “Because of uncertainty about mechanisms linking glucocorticoid (GC) exposure and diabetes mellitus (DM), we fitted 7 conventional models, each using a different representation of time-varying GC exposure . . . Models 5 and 6 used continuous time-varying measures of cumulative dose until a given time point, either in the last year or since study entry, respectively. Model 7 categorized cumulative dose since cohort entry, with cutoff points (based on quartiles) at 0, 960, 3,055, and 7,300 mg PED [prednisolone equivalent dose].”^48^

Larivée and colleagues report: “The aim of this study was to describe the challenge of studying the risk of VTE [venous thromboembolism] among first-time users of drospirenone-containing COCs [combined oral contraceptives] in a healthcare database and assess the risk among first-time users and restarters . . . The first-time user cohort included all women aged 16-45 years who received a first ever prescription of drospirenone- or levonorgestrel-containing COCs between May 2002 and March 2015. The restarter cohort included those who were restarting a COC after a period of non-use of ≥6 months.”^40^

### RECORD-PE item 7.1.f

Use of any comparator groups should be outlined and justified.

#### Explanation

Confounding by indication has also been called an “intractable” bias in epidemiology,^49^ because the choice of treatment is guided foremost by the risk of a particular outcome; hence this item has particular relevance to pharmacoepidemiology. This bias might lead to strong confounding, perhaps greater than that arising from associations due to underlying common causes. Moreover, the degree of confounding by indication is difficult to assess, because it is based on an expected prognosis, and that expectation is formed in the mind of an individual health professional dealing with an individual patient.

An appropriate choice of a comparator treatment is key to reducing confounding by indication or severity (see RECORD-PE item 4.a). If there is no comparator group or cohort, authors should state why. Clear description of the use and justification of comparator groups is needed for the assessment of the potential for confounding by indication or severity. Comparators might include alternative drug exposures for the same indication, differing time windows for the same drug exposures, use of historical comparators, unexposed periods, or unexposed individuals. In the absence of randomisation, confounding (by indication) deserves close attention. Therefore, researchers can use more than one comparison group and make inferences based on whether the estimate of association changes in response to better control of confounding (eg, whether an odds ratio based on an active comparator differs with or without adjustment for confounders), and these analyses should be reported in the published paper. Historical active comparator groups can be assembled from routinely collected health data for single arm studies or when a contemporaneous active comparator is not available; any of these approaches should be clearly reported.

#### Examples of consideration of comparator drugs

In a study examining the association between use of antidepressants and pregnancy/offspring outcomes, Sujan and colleagues dealt with confounding by indication by using exposures during non-relevant gestational periods: “To explore whether intrauterine exposure was specifically associated with outcomes over and above maternal depression treatment around the time of pregnancy, associations for maternal first-trimester antidepressant dispensations were compared with associations for dispensations before pregnancy, while adjusting for measured pregnancy covariates, maternal covariates, and paternal covariates . . . Additionally, the fit of models that included separate parameters for before-pregnancy dispensations and first trimester dispensations were compared with models that included 1 parameter for both dispensation windows. Paternal first trimester antidepressant dispensations were used as a negative control to further explore the role of familial confounding.”^34^

Filion and colleagues report: “Our primary reference group was patients receiving treatment with combinations of oral antidiabetic drugs. With guidelines recommending that incretin-based drugs be used as second-line or third-line therapy, the use of this reference group both reduced potential confounding by indication and provided a clinically relevant treatment comparison.”^50^

### RECORD-PE item 7.1.g

Outline the approach used to handle individuals with more than one relevant drug exposure during the study period.

#### Explanation

In a cohort study comparing the incidence of an adverse reaction between two or more drug exposures, the method of handling individuals who receive multiple drugs at the start of their exposure period (or drug 1 initially, followed by drug 2) should be described to enable readers to interpret findings (see also RECORD-PE items 7.1.d and 7.1.e). Some studies exclude patients who, according to the prescription or dispensing record, receive more than one treatment at the same time at cohort entry, since attribution of risk is difficult. Censoring is used most often if more than one treatment is experienced during follow-up (see below). Alternatively, exposure to more than one treatment (eg, while switching from an old treatment to a novel treatment) can be handled through time varying exposure, whereby each patient’s person time is segmented, based on the dispensing record, with appropriate methods for handling time dependent confounding (eg, marginal structural models, g estimation). The approach taken by authors should be reported transparently, including defining risk attribution models and lag periods.

#### Examples of handling multiple drug exposures

Use of time varying exposures was reported clearly by Xue and colleagues in an international pharmacovigilance study of women with postmenopausal osteoporosis treated with denosumab: “because a large proportion of new Prolia users may have been previously treated with a bisphosphonate, a new-user designwhich mitigates biases associated with previous treatments, if adopted, will be based on a very small number of patients. Also, patients with osteoporosis tend to switch treatments over time, so an open-cohort design combined with an ‘as treated’ analysis was selected to account for time-varying medication exposure.”^51^

Wong and colleagues report: “Based on age within five years, sex, and calendar year at use, we matched one clarithromycin user with one or two amoxicillin users. In both groups we excluded patients who had been prescribed clarithromycin up to four years before the date of first antibiotic prescription during the observation period. However, amoxicillin users could be classified as using clarithromycin at a later date. The observation period commenced from the date of the first antibiotic prescription (index date) and ended at the earliest occurrence of the outcome, death, subsequent use of clarithromycin or amoxicillin, or end of study (31 December 2012).”^52^

## Methods (data sources)

### RECORD-PE item 8.a

Describe the healthcare system and mechanisms for generating the drug exposure records. Specify the care setting in which the drug(s) of interest was prescribed.

#### Explanation

The type of healthcare system, the characteristics of patients for whom drug data are available, and the extent to which patients are reimbursed for prescription drugs could affect the likelihood of drug use, and the likelihood of a record of drug use being included in the study data—for example, formulary restrictions could preclude the use of drugs. Understanding this context will be important for interpretation of generalisability or understanding the limitations in availability of drugs in different settings. For example, although Canada has a government funded, universal healthcare system, some provinces reimburse all prescription drug costs, whereas other provinces only cover drug costs in specific age groups or in people with low incomes who are receiving social assistance. In this second group of provinces, supplemental private insurance might be common among non-covered groups, and therefore drug records could be incomplete in provincial health administrative data. This missing information could result in partial ascertainment, because complete prescription records are available only for certain patients. Left truncation also could create bias if public insurance coverage is available only for older patients. Therefore, characteristics of the health system and context of drug data collection should be provided.

#### Examples describing the healthcare system within which drugs were prescribed

Larivée and colleagues report: “Restarters of COCs [drospirenone-containing combined oral contraceptives] can also be misclassified as first-time users in UK databases as oral contraceptives are commonly prescribed at family planning clinics (i.e., community contraception clinics, genitourinary medicine clinics, sexual health clinics). In England, approximately 7.9% of women aged under 16 attended a family planning clinic from 2009 to 2010 and 21.5% of women aged 16–19 years visited a family planning clinic from 2008 to 2009. The CPRD [Clinical Practice Research Datalink] only captures prescriptions issued by the general practitioner, and the availability of oral contraceptives at family planning clinics makes the identification of first-time users difficult. To attempt to overcome this issue, we applied several exclusion criteria, such as the exclusion of all women with previous prescriptions for hormonal contraception issued by the general practitioner and those with diagnostic codes indicating previous use of hormonal contraception. In addition, we excluded all women with a diagnostic or referral code indicating previous visits to a family planning clinic any time before cohort entry.”^40^

Khan and colleagues report: “Using unique patient identifiers, stroke patients identified in the registry were linked to the Ontario Drug Benefits Database, which contains information on antihypertensive drug prescriptions, including the quantity and dates of drugs dispensed as well as the number of days supplied from each prescription, for patients ≥65 years of age. Residents may fill prescriptions at any outpatient pharmacy in Ontario with a maximum copayment of $6.11 (Canadian) for each prescription after a yearly $100 (Canadian) deductible. Low income seniors have a $2 (Canadian) maximum copayment with no yearly deductible. Using postal codes, patients in the registry are also linked to data from the 2006 Canada Census to determine median neighborhood income.”^53^

## Methods (bias)

### RECORD-PE items

No items specific to the RECORD-PE guidelines are needed in addition to the RECORD and STROBE items.

#### Explanation

Biased studies are characterised by systematic error in observed associations, and readers need to understand the approaches taken to manage bias in order for them to judge whether the results are biased. Several potential biases are likely to arise within pharmacoepidemiological studies, and could be more prominent when routinely collected data are used. Recent papers describing use of triangulation could be helpful in discussions about bias.^54^ The ROBINS-I (risk of bias in non-randomised studies of interventions) tool might also help to focus discussions of bias.^55^ We list below some potential sources of bias in pharmacoepidemiological studies that should be reported.

- Confounding in pharmacoepidemiological analyses might be addressed by design or analytical approaches.^56^ Examples of designs or design features include the use of self controlled case series, instrumental variables, regression discontinuity design, and active comparators. Examples of analytical approaches include the use of multivariable regression analysis or propensity scores, although these approaches will not guarantee absence of confounding. The study design or analytical approach used to address confounding should be reported, and the authors should note in the discussion the extent to which these methods potentially addressed or failed to address the risk of confounding. If more than one method was used, authors should make clear which approach was the main analysis and which were sensitivity analyses.
  For example, in studies applying the propensity score methodology to handle baseline confounding, the method of propensity score estimation should be reported (eg, logistic regression). These scores can be used to adjust for baseline confounding by several methods: propensity score matching, propensity score stratification, covariate adjustment, and inverse probability of treatment weighting.^57^ The specific approach (or approaches) used should be clearly described, together with any attempts to assess the similarities of the resulting treated and untreated groups for each baseline variable.^58^ ^59^ If investigators have used trimming approaches, they should discuss the resulting number of excluded participants. In particular, high dimensional proxy adjustment based on propensity score methods has been reported to reduce residual confounding in studies using claims data and if this approach is used, it should be described.^60^ Lists of empirically identified potential confounders should be reported in online appendices.^39^ If other approaches (such as instrumental variables) were used, these should be clearly described in the publication with similar detail to those outlined for propensity scores.^61^
  Of particular relevance when considering confounding in studies of drug treatment is the type of treatment effect that the non-interventional study is attempting to measure. Types include the intention-to-treat effect (the comparative effect of being assigned to treatment strategies at baseline carried forward, regardless of whether study individuals adhere to the specific treatment) and the as-treated effect (the comparative effect of a drug while it is actually used). In cases in which observational studies based on routinely collected data are designed to emulate a hypothetical or real trial, authors should clearly specify any relevant existing or hypothetical trial that is being emulated. For studies that allow individuals to switch drug treatments as part of the analysis, the role of potential time varying confounders should be considered and reported in the text, along with details of any complex statistical methods applied (such as inverse probability weighting of marginal structural models). For example, in their study of the effect of aspirin on cardiovascular mortality,^62^ Cook and colleagues include a directed acyclical graph of the hypothetical relation between aspirin use, cardiovascular death, and intervening cardiovascular events to show the role of non-fatal cardiovascular events as potential time varying confounders or intermediate steps in the association between aspirin use and cardiovascular mortality.
- Confounding by health status (healthy initiator bias or depletion of susceptibles) can be dealt with at the design stage by aligning the start of observation with treatment initiation (the active comparator new user design; see RECORD-PE item 4.a). The reasoning behind the decision to use such a design feature as well as the extent to which selection bias was or was not addressed should be discussed.
- Information and selection bias due to potential misclassification of drug exposure by prescription or redemption records can be dealt with in a sensitivity analysis that includes different definitions of exposures (eg, when different algorithms are used to define duration of prescribing episodes^63^). An alternative approach is to include only people with more than one prescription or redemption over a given period (eg, within six months), because those individuals with just one prescription or redemption might never have used the treatment. Use of different washout periods to define new episodes of treatment could also affect the interpretation of data. Each issue should be clearly described and discussed in reports of pharmacoepidemiological studies based on routinely collected health data.

## Methods (statistical methods)

### RECORD-PE item 12a

Describe the methods used to evaluate whether the assumptions have been met.

#### Explanation

In reporting all study designs, authors should discuss whether the underlying study assumptions have been met. A failure of the assumptions being met could undermine the methods used. Determining whether the methods used were appropriate, given the data, is important for readers to understand whether the resulting analysis requires further consideration. A pharmacoepidemiological example is the use of self controlled studies, in which participants act as their own controls, and include the case crossover design and self controlled case series studies.^64^ When applying the self controlled case series method, several assumptions must be met to obtain valid and unbiased estimates,^65^ for instance, exposure to the drug of interest must not be influenced by a previous outcome event.^66^ ^67^ Authors should specify clearly how the assumptions of self controlled or other study designs were evaluated. Detailed guidance on the conduct and reporting of self controlled case series are beyond the scope of these guidelines, but are currently being developed by the SCOPE (self controlled crossover observational pharmacoepidemiology) initiative.^68^ All reports should explain any assumptions that were not evaluated or not met. Discussions should also address the possibility of time related bias (eg, immortal time bias),^69^ if these are likely to be a problem.

#### Example on testing study assumptions

Wilson and colleagues report: “We graphed the number of combined endpoint events in the days before and after vaccination. In the self-controlled case series model, the date of vaccination serves as the index date for exposure for each patient. Previous studies have identified that children are at increased risk for systemic reactions at different times from 5–14 days after vaccination . . . Because *a priori* we did not know with certainty the time period following vaccination for which there would be an increased risk of our combined endpoint, we modified the standard self-controlled case series approach by looking for an elevation in risk during each post-vaccination day up to day 17 . . . We then classified days 20–28 as unexposed, establishing a washout period in between the exposed and unexposed periods . . . When multiple events occurred to a given individual, the first occurrence of the composite outcome in the post-vaccination period was used (*e.g.,* someone attending the ER [emergency department] who was then admitted would have one event counted in that period). The relative incidence rate of the composite endpoint during the exposed period compared with the unexposed period was analyzed using a fixed effects Poisson regression model. This model included a term for exposure period and a term for patient, thereby allowing each individual to serve as his or her own control and accounting for intra-individual correlation. An offset term was also included to account for the differing durations of the exposed and unexposed periods.”^70^

### RECORD-PE item 12.b

Describe and justify the use of multiple designs, design features, or analytical approaches.

#### Explanation

As discussed in RECORD-PE item 4.a, the use of multiple designs or design features in the same report is a commonly used strategy in pharmacoepidemiological studies to assess the potential for bias and residual confounding. If authors have used multiple approaches to analysis, these should be clearly outlined for readers to assess strengths and limitations. The authors also should state clearly how they approached reproducibility across different databases, including issues such as variability in coding and healthcare systems. If authors used a common data model^71^ (see the second example^72^ below) to analyse data across different data sources, they should describe this and specify which common data model they used. If any data pooling across data sources was done, the approaches used should be described.

#### Examples describing each design, design feature, or analytical approach

Wong and colleagues report: “We used Poisson regression to estimate the rate ratios for clarithromycin users compared with amoxicillin users during current, recent, and past use . . . For the self-controlled case series analysis, we estimated incidence rate ratios using conditional Poisson regression, comparing the rate of events during risk windows with the rate during baseline periods . . . we also performed a post hoc case crossover analysis, which is not vulnerable to this limitation of the self controlled case series. The case crossover design is applied for studies investigating the association between transient drug use and outcome with abrupt time of onset. We estimated odds ratios using conditional logistic regression, comparing drug use before the event (current period) with that at other earlier control periods within patients.”^52^

But and colleagues report: “The individual-level data from the five cohorts were standardised by each research partner locally using the common data model. We then conducted centralised analyses by uploading the unified data to a server at Statistics Denmark, where for each cohort we constructed the individual-level dataset to assess insulin exposure and other variables in exactly the same way. We employed a semi-aggregate level approach to combine the datasets, which were tabulated by cancer site as the number of cancer cases and person-years aggregated by categorical variables.”^72^

## Methods (data access and cleaning methods)

### RECORD-PE items

No items specific to the RECORD-PE guidelines are needed in addition to the RECORD items.

#### Explanation

RECORD states that “authors should provide information on the data cleaning methods used in the study.”^1^ This information is particularly important for pharmacoepidemiological studies, because the preparation of drug exposure data is complex and reflects serial assumptions that are typically not disclosed. Therefore, data cleaning extends substantially beyond the removal of outlying values. When data require preparation for analysis (eg, conversion of raw prescription data to exposed and unexposed episodes of person time), authors should be transparent about the steps undertaken in cleaning the data. These steps might include decisions on deriving start and stop dates; and assumptions made when instructions on administration instructions provide for flexibility (eg, prescriptions as needed), when prescriptions are overlapping, and when clinically implausible values are encountered.

## Results

### RECORD-PE items

For the results section, no items specific to RECORD-PE are needed in addition to the earlier STROBE and RECORD recommendations.

#### Explanation

STROBE guidelines recommend that researchers report the number of individuals included at each stage of the study, including reasons for exclusion.^18^ The RECORD guidelines further underscore the importance of reporting how results were filtered based on data quality, availability, and linkage.^1^ Use of a flow diagram to illustrate the selection of the study population is encouraged by both STROBE and RECORD—note that this diagram is distinct from the study design diagram discussed in RECORD-PE item 4.b. A high level of transparency is equally important in pharmacoepidemiological studies in which additional eligibility criteria are often used (eg, based on indications for use, washout periods, and lag periods), adding further complexity to the selection process. Researchers should report the number of participants included at all stages of the study, including the analysis stage and for analyses performed to assess different objectives (eg, subgroup and sensitivity analyses).

Pharmacoepidemiological studies that examine adverse drug events or reactions should report whether and how researchers assessed or validated the outcome on the individual case level (eg, through record review by a specialist blinded to the exposure(s) under study, in order to try and rule out other more likely causes of the event). This process should be clear from the text, a table, or a flowchart describing how many events were considered to be caused by the study drug(s) after record review. Presenting numbers of potential cases that lacked sufficient data to be classified as non-cases or definite cases (and were assigned a final status such as possible or uncertain) is also encouraged. In an article by Kaye and colleagues on the risk of liver injury associated with use of oral antimicrobials, figure 1 provides a good example.^73^ Clear delineation of the selection process facilitates critical appraisal, applicability, and reproducibility of the study findings.

Regarding results from descriptive analyses, STROBE recommends that authors present detailed data on the distribution of demographic, clinical, and social variables, including the number of participants with missing data. Missing data are frequently encountered in pharmacoepidemiological studies based on routinely collected data. In studies using routinely collected data, we might not know whether there is non-recorded or unmeasured information on diagnoses, symptoms, and management. Cohort studies also should provide summary measures of follow-up time. For studies based on routinely collected data, RECORD does not include additional items.^1^ However, in terms of clinical variables, pharmacoepidemiological studies should report the distribution of indications for the drug of interest. It is also advised that authors summarise person time on and off drug exposure, including the sensitivity of “at risk” periods to different definitions of risk attribution if appropriate. Furthermore, in the case of time varying variables, which are increasingly used in pharmacoepidemiology, authors should consider reporting the number and characteristics of individuals with time varying data.

RECORD-PE supports the STROBE recommendations for presentation of outcome data, main results, and other analyses.^18^ Thus, researchers should report the number of events or summary measures of outcomes (or exposures in case-control studies), unadjusted and adjusted estimates and their precision, confounder variables adjusted for, category boundaries when continuous variables are categorised, absolute measures of risk for a meaningful time period (if relevant), and other analyses performed (including subgroup, interaction, and sensitivity analyses). Authors should present the results determined using the different approaches, which could include conventional methods and more complex approaches.

If multiple approaches have been used in an attempt to account for confounding (eg, matching and adjustment), the results of all methods should be presented and any differences discussed. It is advisable to present descriptive results showing covariate distribution (number and percentages) in exposure groups before propensity score matching, as well as the distribution after propensity score matching if appropriate. Authors should explicitly state whether an analysis was prespecified or post hoc. Researchers also are advised to report in detail the results of analyses used to explore and handle missing data, which are frequently encountered in pharmacoepidemiological studies based on routinely collected data.

## Discussion (limitations)

### RECORD-PE item 19.1.a

Describe the degree to which the chosen database(s) adequately captures the drug exposure(s) of interest.

#### Explanation

Authors should report whether the drug exposure in question could be ascertained by an alternative source, if not fully captured in the database used for the study. Some of the explanation outlined in RECORD-PE item 8.a is also relevant here. An additional issue relates is whether a drug exposure of interest could have been obtained over the counter, and whether such use is captured by the data source.^74^ If not, authors may want to discuss the likely extent of misclassification. A similar issue is that if patients are admitted to hospital for extended periods and the database does not capture inhospital dispensing of drug treatments, misclassification also might occur.^69^ ^75^ Authors should also discuss whether the database is likely to have had information on diagnoses, symptoms, and management, and discuss the implications for study findings.

#### Examples on adequacy of capture of drug exposure in database

Weinstein and colleagues report: “This analysis was restricted to prescription use of paracetamol and ibuprofen, and it is unknown whether these results would generalize to non-prescription exposures. There are several reasons for a GP [general practitioner] to prescribe these medications in the CPRD [Clinical Practice Research Datalink], including record keeping and giving the patient access to the medication at a lower cost because the patient qualifies for free filling of prescriptions. In addition, those using these medications chronically may need larger quantities than typically available over the counter. Thus, it is likely that, by relying on prescriptions, we skewed our study population toward elderly subjects with chronic conditions who may also be at the low end of the economic spectrum.”^76^

Suissa reports: “In our illustration, the naive approach that does not account for the immeasurable hospitalized time during the 30-day period prior to the index date estimated a significant 40 percent reduction in mortality associated with a prescription of inhaled corticosteroids during this period. However, there were 806 cases (deaths) that had been hospitalized during this same 30-day period and that were considered unexposed by this analysis since they did not receive a prescription. These cases had spent 16.2 out of the 30 days in the hospital, time during which they could not receive outpatient prescriptions, compared with 8.8 days for the corresponding 253 such controls. In fact, 190 of these 806 cases (24 percent) had spent the entire 30-day period in the hospital, compared with seven of the 253 controls (3 percent), and could not possibly have received any prescription at all.”^69^

## Discussion (interpretation)

### RECORD-PE item 20.a

Discuss the potential for confounding by indication, contraindication, or disease severity or for selection bias (healthy adherer or sick stopper) as alternative explanations for the study findings when relevant.

#### Explanation

As discussed in the methods section, confounding by indication is a major issue in interpreting the findings of pharmacoepidemiological studies, beyond studies of routinely collected data in general. Particularly in the case of insurance or billing data (such as health administrative data), identification of study participants, drug exposures, confounders, and outcomes are based on coded data. Little or no information might be available to describe the indication for drug treatment, personal preferences and values of the patient and prescriber, any potential contraindications to use, or disease severity, all of which could confound the association between the drug and the outcome of interest. Even in clinical data (such as data from electronic health records), the indication or contraindication for drugs might not be recorded or might be contained in free text fields, and not accessible to investigators using these data for pharmacoepidemiological research. Important confounding variables thus might not be available for investigators or readers of the research report. Therefore, to the best of their ability, authors should report such potential confounding in the routinely collected health data and how the confounding was managed overall.

We recommend the inclusion of a clear statement in the conclusions (or other discussion section) to explain whether the results could be explained by confounding by indication. Such a statement would help reduce misguided decision making and increase the trustworthiness of the evidence and its interpretation. This statement could report any post hoc analyses designed to evaluate the robustness of the finding and alternative explanations—for instance, to evaluate whether patients in different exposure groups were likely to have been prescribed the drugs for similar conditions. If moving of an individual to another practice or insurance database cannot be tracked, authors should clearly outline whether that individual’s person time is unique (that is, only considered from the new registration date) and discuss the effect of missing past exposures or events on the study findings. A histogram of people leaving for reasons other than death by year of age also might be useful, as well as the age distribution of people entering the population.

Further, as per RECORD-PE item 12.1.b, authors should include explicit consideration of results from different approaches when they have used multiple designs, design features, or analytical approaches. This information is especially pertinent if such efforts yield inconsistent results and thus guidance on interpretation is needed.

#### Examples on alternative explanation for findings

Sujan and colleagues report: “The findings from the present study should be considered in light of several limitations. First, and most important, observational designs such as these cannot fully rule out all sources of confounding. In particular, like other register-based approaches, this study could not comprehensively assess maternal depression or its severity, nor could it compare different antidepressant treatment regimens. Thus, associations could have been influenced by confounding by antidepressant indication . . . the study used multiple designs to address this limitation, each of which could help rule out some but not all sources of confounding, to provide complementary evidence. For example, sibling comparisons ruled out all stable confounders (*e.g*., chronic maternal depression), but that design may not have been able to account for confounding from maternal depression that varied across pregnancies. Thus, the within-family associations with preterm birth may plausibly be driven by unmeasured time-varying maternal depression rather than by antidepressant use.”^34^

Filion and colleagues report: “Our study was designed to examine the impact of drug formulary restrictions on the validity of pharmacoepidemiologic studies using the example of fluticasone/salmeterol combination therapy. We found that the implementation of these restrictions had a profound effect on drug utilization, with the policy resulting in an important decrease in the rates of prescription and of new use of fluticasone/salmeterol. These prescription changes resulted in channeling and confounding by indication, with new users of fluticasone/salmeterol having a significantly higher crude rate of hospitalization for respiratory causes during the restricted period (crude HR [hazard ratio] = 1.41, 95%CI [confidence interval] = 1.32, 1.51) because of the presence of more severe underlying respiratory disease. Adjustment for potential confounders attenuated and reversed the association, with new users during the restricted period having a significantly lower rate of hospitalization for respiratory causes compared with those during the liberal period (fully adjusted HR = 0.78, 95%CI = 0.73, 0.83). These results suggest that drug formulary restrictions can result in substantial and unexpected confounding by indication that threatens the validity of study results. These results also suggest that adjusting for patient demographic and clinical characteristics is insufficient to account for channeling because of formulary restrictions. Consequently, such restrictions must be considered in the design and analysis of pharmacoepidemiologic studies.”^77^

Schneeweiss and colleagues report: “Aprotinin rather than aminocaproic acid was used in sicker patients, and the modest reduction in the relative mortality estimates after the control of confounding by covariates is consistent with the hypothesis of confounding on the basis of indication. Multivariate analyses resulted in weaker associations between aprotinin and death than those reported in unadjusted analyses (unadjusted relative risk, 1.83; adjusted relative risk, 1.64). Matching according to propensity score permitted us to control for an additional 10 covariates in a highly selected cohort, which further reduced the relative-risk estimate.

“Our analyses were adjusted for some, but not all, covariates typically included in risk-prediction scores for patients undergoing CABG [coronary artery bypass grafting]. However, we adjusted for many covariates not typically included, and controlling for proxies of confounders results in control of the confounders themselves if the proxies capture the relations with the true confounding variable, exposure, and outcomes. Our joint adjustment for 41 characteristics before CABG was performed resulted in the prediction of in-hospital death that is as good as that from widely accepted clinical risk-prediction models for patients undergoing CABG. Prediction was almost identical for patients receiving aprotinin and for those receiving aminocaproic acid.”^78^

## Discussion of the RECORD-PE checklist

The complete and accurate reporting of research is an ethical requirement endorsed by leading declarations and recommendations internationally.^79^ ^80^ The RECORD-PE guidelines have been developed to meet an identified need and are designed to improve the reporting of pharmacoepidemiological studies undertaken using routinely collected data. They are an extension of the STROBE and RECORD statements and should be used in conjunction with the existing guidelines.^1^ ^18^ ^81^ RECORD-PE represents a minimum standard of reporting and complements a recent set of comprehensive methodological and reporting items created with the aim of making pharmacoepidemiological research more reproducible.^22^ Better reporting is a prerequisite of replication, but replication requires much more detail. However, RECORD-PE also guides authors on the transparency of reporting and helps readers to understand strengths and potential limitations of the work. Therefore, RECORD-PE represents a minimum standard of reporting for pharmacoepidemiological studies undertaken using routinely collected health data.

### Limitations

We consulted widely in the creation of these guidelines, including international experts in pharmacoepidemiology, journalology, editors, and policy makers. Despite wide consultation, we may have missed some key points. In addition, members of our guideline development working committee were primarily from western Europe and North America.

Pharmacoepidemiology is a rapidly changing field with frequent new methodological developments, reflecting the growth of big data, the development of integrated or distributed data systems, and the innovative approaches being developed to reduce bias associated with the use of non-randomised data to assess drug effects. Increasing collaborative approaches across traditional geographical and data source boundaries are leading to new discoveries for patient benefit in pharmacovigilance and pharmacoepidemiology to overcome problems such as sample sizes insufficient to detect less common safety signals. We have included some recent developments briefly in this article, for example, the use of common data models. We recognise that these guidelines will need to be updated to encompass new developments.

The RECORD-PE guidelines are an extension of the STROBE guidelines for non-interventional research, hence the focus of these guidelines is largely on non-interventional research in pharmacoepidemiology. There is much discussion in the literature about whether the term “observational” should be used as opposed to the term “non-interventional studies,” because all studies involve observation. For RECORD-PE, we have continued with the term “observational” in the title because RECORD-PE is an extension of RECORD which, in turn, is an extension of the STROBE guidance.^1^ ^18^ We have briefly mentioned the use of pragmatic trials using routinely collected health data in the RECORD-PE guidelines; however, the increasing use of pragmatic randomised controlled trials (and particularly the development of registry based trials and trials within cohorts^82^ ^83^) will likely necessitate expansion of the currently available guidance with both RECORD and CONSORT as guiding documents.

### Conclusions of the RECORD-PE checklist

The RECORD-PE statement aims to extend existing STROBE and RECORD guidelines providing guidance for the reporting of pharmacoepidemiological studies using routinely collected data. It aims to enable readers to understand what was planned, what was done, and what was found in the research. This essential information is critical for users of research to optimally interpret the findings, including their strengths and limitations. Poor research reporting hampers the use of research findings and is an important component of research waste.^84^ We anticipate that with increasing use of the RECORD-PE guidelines by researchers and endorsement and adherence by journal editors, the reporting of pharmacoepidemiological research undertaken using routinely collected health data will improve. The improved transparency and accuracy will benefit the research community, and ultimately improve patient care.

## Web extra

Extra material supplied by authors

**Supplementary table 1:** Glossary of terms

**Supplementary table 2:** RECORD-PE checklist

## Notes

Contributors: SML and EIB conceived and designed the study, carried out the statistical analysis, had full access to all the data in the study, take responsibility for the integrity of the data and the accuracy of the data analysis, and are guarantors. All authors acquired data, analysed and interpreted data, drafted the manuscript, and critically revised the manuscript for important intellectual content. The corresponding author attests that all listed authors meet authorship criteria and that no others meeting the criteria have been omitted.

Provenance and peer review: Not commissioned; externally peer reviewed.
