# Supplementary material for: The reporting of studies conducted using observational routinely collected health data statement for pharmacoepidemiology (RECORD-PE)
Source: BMJ. 2018 Nov 14;363:k3532. doi: 10.1136/bmj.k3532 (PMC6234471; doi:10.1136/bmj.k3532)
Supplement: Supplementary file 1 — Supplementary table 1: Glossary of terms [file lans044971.ww1.pdf]

**Supplementary Table 1. Glossary of terms**

| Term | Definition |
|---|---|
| Absolute risk | "Risk in a population of exposed persons; the probability of an event affecting members of a particular population (e.g. 1 in 1,000). Absolute risk can be measured over time (incidence) or at a given time (prevalence)"[1] |
| Active comparator | A comparison intervention/treatment considered to be effective (or active) by health care providers.[2] |
| Administrative databases | "Databases storing information routinely collected for purposes of managing a health-care system" [3] |
| Adverse drug reaction | "An adverse drug event that is judged to be caused by the drug."[4] |
| Bias | "Any systematic (rather than random) error in a study." [4] |
| Case-control study | "Epidemiological design comparing previous exposure to a risk factor of interest (e.g. Use of a drug) or the presence of a characteristic in a group of subjects presenting a given event (the cases), to that in a group not presenting this event (the controls)" [5] |
| Channeling bias | An allocation bias, which occurs when drug therapies with similar indication are prescribed to groups of patients with prognostic differences.[4 6] |
| Clearance (Drug) | "The proportion of the apparent volume of distribution that is cleared of drug in a specified time. Its units are volume per time, such as liters per hour. The total body clearance is the sum of clearances by different routes, for example renal, hepatic, pulmonary, etc."[4] |
| Cohort study | "Studies that identify defined populations and follow them forward in time, examining their frequencies (e.g., incidence rate, cumulative incidence) of disease. Cohort studies generally identify and compare exposed patients to unexposed patients or to patients who receive a different exposure." [4] |
| Comparative Effectiveness Research (CER) | "The generation and synthesis of evidence that compares the benefits and harms of alternative methods to prevent, diagnose, treat, and monitor a clinical condition or to improve the delivery of care. The purpose of CER is to assist consumers, clinicians, purchasers, and policy makers to make informed decisions that will improve health care at both the individual and population levels." [7] |
| Confidence Interval | "a range of values within which the true population value lies, with some probability." [4] |
| Confounding by indication | Is the phenomenon whereby "the underlying diagnosis or other clinical features that affect the use of a certain drug are also related to the outcome under study." [4] |

| | |
|---|---|
| Confounder | "A variable other than the risk factor and outcome variable under study that is related independently both to the risk factor and to the outcome and not on the causal pathway between the exposure and the outcome. A confounder can artificially inflate or reduce the magnitude of association between an exposure and outcome." [4] |
| Cost - identification analysis | "Enumerates the costs involved in medical care, ignoring the outcomes that result from that care." [4] |
| Covariate | "Often used simply as an alternative for explanatory variable, but perhaps more specifically to refer to variables that are not of primary interest in an investigation, but are measured because it is believed that they are likely to affect the response variable and consequently need to be included in analyses and model building." [3] |
| Cross-sectional study | A study which aims to "Examine exposures and outcomes in populations at one point in time; they do not assess temporal relationships."[4] |
| Cumulative dose | "In medicine, the total amount of a drug or radiation given to a patient over time; for example, the total dose of radiation given in a series of radiation treatments." [8] |
| Defined daily dose (DDD) | "The usual daily maintenance dose for a drug for its main indication in adults." [4] |
| Detection bias | "An error in the results of a study due to a systematic difference between the study groups in the procedures used for ascertainment, diagnosis, or verification of disease." [4] |
| Drug utilization | "As defined by the World Health Organization (WHO), is the 'marketing, distribution, prescription and use of drugs in a society, with special emphasis on the resulting medical, social, and economic consequences.' " [4] |
| Effect-modification | "Occurs when the magnitude of effect of a drug in causing an outcome differs according to the levels of a variable other than the drug or the outcome (e.g., sex, age group). Effect modification can be assessed on an additive and/or multiplicative scale." [4] |
| Effectiveness (drug) | "A study of whether, in the usual clinical setting, a drug in fact achieves the effect intended when prescribing it." [4] |
| Efficacy (drug) | "A study of whether, under ideal conditions, a drug has the ability to bring about the effect intended when prescribing it." [4] |
| Efficiency (drug) | "Is a study of whether a drug can bring about its desired effect at an acceptable cost." [4] |
| Half - life (T 1/2) | "The time taken for the drug concentration to decline by half. Half -life is a function of both the apparent volume of distribution and clearance of the drug." [4] |

| | |
|---|---|
| Hazard ratio | "A measure of how often a particular event happens in one group compared to how often it happens in another group, over time. A hazard ratio of one means that there is no difference in the outcome between the two groups. A hazard ratio of greater than one or less than one means that outcome was more or less likely in one of the groups." [8] |
| Healthy Initiator Bias | "Involves either (i) the selective initiation of preventive treatments among healthy and health-conscious patients, who through the effects of their healthy lifestyle, are also at decreased risk of a number of adverse health outcomes, or (ii) the selective channeling of treatments away from frail individuals, who are at an increased risk of adverse outcomes. Under both scenarios, the healthy initiator bias will lead to spurious associations, where the beneficial effect of a given drug will be exaggerated." [9] |
| Immortal Time Bias | Immortal time refers to a period of follow-up during which, by design, death or the study outcome cannot occur. Bias is introduced when this period of "immortality" is either misclassified with regards to treatment status or excluded from the analysis. Immortal time bias is problematic because it biases the results in favour of the treatment under study by generating a spurious survival advantage to the treated group. [10] [11] |
| Incidence rate | A measure of how frequently the disease occurs. Specifically, it is the number of new cases of the disease which develop over a defined time period in a defined population at risk, divided by the number of person-years in that population at risk. |
| Information bias | "An error in the results of a study due to a systematic difference between the study groups in the accuracy of the measurements being made of their exposure or outcome." [4] |
| Interrupted Time Series | "A design in which a string of consecutive observations is interrupted by the imposition of a treatment to see if the slope or intercept of the series changes as a result of the intervention." [12] |
| Knowledge Synthesis | A dynamic and iterative process that includes the synthesis, dissemination, exchange and ethically-sound application of knowledge to improve the health of a population, provide more effective health services, and strengthen the healthcare system.[13] |
| Matching | "Sometimes synonymous with blocking, sometimes more specific to imply blocks in which units are exactly equal (rather than just similar) on a matching variable." [12] |
| Misclassification Bias | Also known as information bias, is the error resulting from classifying study subjects incorrectly exposed or diseased [4] |
| Nsaids | "Non-steroidal anti-inflammatory drugs (nsaids) are among the most commonly used drugs and are mainly used to alleviate pain and inflammation." [14] |
| Nearest Neighbour Matching | "In its simplest form, 1:1 nearest neighbor matching selects for each treated individual *i* the control individual with the smallest distance from individual *i*." [15] |

| | |
|---|---|
| New User Design | A design that identifies all patients in a defined population who start a course of treatment with the study medication, with restriction of participants to those with a minimum period of non-use and analysis to persons under observation at the start of the current course of treatment. [16] |
| Non - differential misclassification | Misclassification whereby "The misclassification of one variable does not vary by the level of another variable. Non -differential misclassification usually results in bias toward the null." [4] |
| Observational study | "(Or non -experimental studies) are studies in which the investigator does not control the therapy, but observes and evaluates the results of ongoing medical care. The study designs that are used are those that do not involve randomization, such as analyses of secular trends, case –control studies, and cohort studies." [4] |
| Pharmacodynamics | "The study of the relationship between drug level and drug effect. It involves the study of the response of the target tissues in the body to a given concentration of drug." [4] |
| Pharmacoepidemiology | "The scientific discipline of studying drug effects in populations" [17] |
| Pharmacovigilance | "The science and activities relating to the detection, assessment, understanding and prevention of adverse effects or any other drug-related problems." [17] |
| Poisson regression | "A method for the analysis of the relationship between an observed count with a Poisson distribution and a set of explanatory variables." [3] |
| Population | "In statistics this term is used for any finite or infinite selection of 'units', which are often people but may be, for example, institutions, events, etc." [3] |
| Pragmatic Trial | "Trials primarily designed to determine the effects of an intervention under the usual conditions in which it will be applied." [18] |
| Prescription-event monitoring (PEM) | "Pharmacoepidemiological study in which a cohort of users of a medicine is identified from prescriptions and followed-up for a defined period (often 6–12 months) so as to identify all adverse events occurring in the early post-treatment period. The data are potentially useful for detecting signals of unexpected effects and/or to further study known or potential safety issues." [17] |
| Propensity score | "An approach to controlling for confounding that uses mathematical modeling to predict exposure based on observed variables, and uses the predicted probability of exposure as the basis for matching or adjustment." [4] |
| Protopathic bias | "Interpreting a factor to be a result of an exposure when it is in fact a determinant of the exposure, and can occur when an early sign of the disease under study led to the prescription of the drug under study." [4] |

| | |
|---|---|
| Registry | "Organized systems that use observational study methods to collect uniform data (clinical and other) to evaluate specified outcomes for a population defined by a particular disease, condition, or exposure, and that serves one or more predetermined scientific, clinical, or policy purposes. Registries can be thought of as both the process for collecting data from which studies are derived, as well as referring to the actual database." [4] |
| Regression Discontinuity Design | "An experiment in which units are assigned to conditions based on exceeding a cut off on an assignment variable" [12] |
| Risk Set | "A term used in survival analysis for those individuals who are alive and uncensored at a time just prior to some particular time point."[3] |
| Selection Bias | "Selection bias is a systematic error in a study that occurs from the process used to identify (select) the study participants, allocate them to study groups and from factors that influence study participation." [19] |
| Self-controlled Case Series | "An epidemiological study design for which individuals act as their own control—ie, comparisons are made within individuals" [20] |
| Sensitivity Analysis | "A set of procedures in which the results of a study are recalculated using alternate values for some of the study's variables, in order to test the sensitivity of the conclusions to altered specifications." [4] |
| Truncated data | "Data for which sample values larger (truncated to the right) or smaller (truncated to the left) than a fixed value are either not recorded or not observed." [3] |
| Washout Period | "More or less complete elimination of the active drug at the conclusion of a period without treatment. A sufficiently long washout period is indispensable for bringing the study subjects back to their baseline status, that is, to assume that the active principle is no longer able to interfere with the proposed measurement. Such free intervals can be planned before inclusion of previously treated subjects, or, in the case of crossover studies, in between treatment sequences being compared." [5] |
| Weighted Cumulative Exposure Model | A model that that combines information about duration, intensity and timing of exposure to estimate the total effect of past exposures using a weighted sum of these exposures, with the weights dependent on time since exposure. [21 22] |

**References**

1. Eu2P. Glossary of terms used in pharmacovigilance and pharmacoepidemiology no date [Available from: https://www.eu2p.org/help/glossary accessed 27 March 2018.

2. Clinicaltrials.gov. Glossary of Common Site Terms: U.S. National Library of Medicine; 2018 [Available from: https://clinicaltrials.gov/ct2/about-studies/glossary accessed 27 March 2018.
3. Everitt BS. The Cambridge dictionary of statistics. 3rd ed. Cambridge, UK: Cambridge University Press 2006.
4. Pharmacoepidemiology. 5th edition ed: John Wiley & Sons 2012.
5. Bégaud B. Dictionary of pharmacoepidemiology. Chichester, UK: John Wiley & Sons 2000.
6. Petri J, Urquhart J. Chenneling bias in the interpretation of drug effects. *Stat Med* 1991;10(4):577-81.
7. IOM (Institute of Medicine). Initial National Priorities for Comparative Effectiveness Research2009.
8. National Cancer Institute. NCI Dictionary of Cancer Terms no date [Available from: https://www.cancer.gov/publications/dictionaries/cancer-terms accessed 27 March 2018.
9. Lund JL, Richardson DB, Sturmer T. The active comparator, new user study design in pharmacoepidemiology: historical foundations and contemporary application. *Curr Epidemiol Rep* 2015;2(4):221-28. Doi: 10.1007/s40471-015-0053-5 [published Online First: 2016/03/10]
10. Suissa S. Immortal time bias in pharmacoepidemiology. *Am J Epidemiol* 2008;167(4):492-9. Doi: 10.1093/aje/kwm324 [published Online First: 2007/12/07]
11. Lévesque L. Problem of immortal time bias in cohort studies: example using statins for preventing progression of diabetes. *BMJ* 2010;340
12. Shadish WR, Cook TD, Campbell DT. Experimental and quasi-experimental designs for generalized causal inference. Belmont, California: Wedsworth Cengage Learning 2002.
13. Grimshaw J. A Guide to Knowledge Synthesis. A knowledge synthesis chapter Ottawa, Canada: Canadian Institutes of Health Research (CIHR); 2010 [updated 2010-04-08. Available from: http://www.cihr-irsc.gc.ca/e/41382.html accessed March 12 2018.
14. Gislason GH, Torp-Pedersen C. Nsaids and the failing heart. *BMJ* 2016;354:i5163. Doi: 10.1136/bmj.i5163 [published Online First: 2016/09/30]
15. Stuart EA. Matching methods for causal inference: A review and a look forward. *Stat Sci* 2010;25(1):1-21. Doi: 10.1214/09-STS313 [published Online First: 2010/09/28]
16. Ray WA. Evaluating medication effects outside of clinical trials: New-User Designs. *American Journal of Epidemiology* 2003;158(9):915-20.
17. Waller P. An Introduction to Pharmacovigilance: Wiley-Blackwell 2010.
18. Thorpe KE, Zwarenstein M, Oxman AD, et al. A pragmatic-explanatory continuum indicator summary (PRECIS): a tool to help trial designers. *J Clin Epidemiol* 2009;62(5):464-75. Doi: 10.1016/j.jclinepi.2008.12.011 [published Online First: 2009/04/08]
19. Rothman KJ. Epidemiology - An introduction. New York: Oxford University Press 2002.
20. Petersen I, Douglas I, Whitaker H. Self controlled case series methods: an alternative to standard epidemiological study designs. *BMJ* 2016;354:i4515. Doi: 10.1136/bmj.i4515 [published Online First: 2016/09/14]
21. Sylvestre MP, Abrahamowicz M. Flexible modeling of the cumulative effects of time-dependent exposures on the hazard. *Stat Med* 2009;28(27):3437-53. Doi: 10.1002/sim.3701 [published Online First: 2009/08/27]
22. Yu D, Peat G, Bedson J, et al. Weighted cumulative exposure models helped identify an association between early knee-pain consultations and future knee OA diagnosis. *J Clin*

*Epidemiol* 2016;76:218-28. Doi: 10.1016/j.jclinepi.2016.02.025 [published Online First: 2016/03/13]
